# Development and Characterization of Silver-Doped Multi-Walled Carbon Nanotube Membranes for Water Purification Applications

**DOI:** 10.3390/membranes12020179

**Published:** 2022-02-02

**Authors:** Muhammad Umar Amjad, Bilal Anjum Ahmed, Faisal Ahmed, Hasan Aftab Saeed

**Affiliations:** Department of Mechanical Engineering (CEME), National University of Sciences and Technology (NUST), Sector H-12, Islamabad 4600, Pakistan; muhammad.amjad18@me.ceme.edu.pk (M.U.A.); faisal.ahmed@ceme.nust.edu.pk (F.A.); hasan.saeed@ceme.nust.edu.pk (H.A.S.)

**Keywords:** multi-walled carbon nanotubes, membranes, silver particles, water purification, impregnated MWCNTs

## Abstract

A unique approach was utilized to develop multi-walled carbon nanotube (MWCNT) silver (Ag) membranes. MWCNTs were impregnated with 1 wt% Ag loading, which resulted in a homogeneous dispersion of Ag in MWCNTs. MWCNTs impregnated with Ag were then uniaxially compacted at two different pressures of 80 MPa and 120 MPa to form a compact membrane. Compacted membranes were then sintered at two different temperatures of 800 °C and 900 °C to bind Ag particles with MWCNTs as Ag particles also act as a welding agent for CNTs. The powder mixture was characterized by FESEM, thermogravimetric analysis, and XRD, while the developed samples were characterized by calculating the porosity of membrane samples, contact angle, water flux and a diametral compression test. The developed membranes showed overall large water flux, while maximum porosity was found to decrease as the compaction load and sintering temperature increased. The mechanical strength of the membranes was found to increase as the compaction load increased. The hydrophilicity of the membranes remained unchanged after the addition of Ag particles. The developed membranes would be useful for removing a variety of contaminants from water.

## 1. Introduction

The need for pure water is rising rapidly in the industrial, agriculture and domestic sectors [1]. Pollutants such as arsenic [2], heavy metal ions, and organic and inorganic impurities [3,4] from various industries are contaminating water on a large scale. Thus, water purification becomes a critical issue to protect the environment and human health [1]. It is therefore the need of the hour to develop water purification techniques that can meet the rapidly rising demand for clean water [5,6]. More specifically, durable, cheap, and reliable water purification technologies are needed to produce purified water from desalinated or brackish water [7].

Nanotechnology has promising potential in the field of water purification. Various nanoparticles (Ag, Fe, Ti and Au) and their oxides have been extensively utilized to control undesired environmental impacts. Among these, Ag nanoparticles are beneficial to limit the growth of viruses and bacteria [8]. Different nanomaterials such as CNTs, zeolites, and metals and their oxides have been extensively used for water purification. Among all these, CNTs stand out because of their exceptional mechanical, electrical, and thermal characteristics. CNTs have been used extensively in the removal of different types of pollutants, such as heavy metal ions (chromium, nickel, zinc, lead and cadmium), radioactive nuclides [7,9,10,11,12,13,14,15,16,17,18,19], and inorganic and organic contaminants [20,21,22,23].

Membrane separation technologies can be generally categorized based on the driving force utilized to generate the separation. The movement of water through the membrane in reverse osmosis (RO) [24] and nanofiltration membrane operations require the applied hydraulic pressure gradient to exceed the osmotic pressure gradient between the input and the filtrate [25]. Forward osmosis, on the other hand, uses an osmotic pressure differential to move water [26].

In recent years, RO water filtration has gained significant importance because of its ability to purify various types of waste- and seawater [27]. RO is now marked as the most significant water purification system for seawater [28]. RO water filtration facilities use a high pressure to filter pollutants, and the membrane plays an important role in this process. The membrane in a RO water purification system acts as an obstacle that only allows water to pass through it, blocking the other impurities present in the water [29]. However, like all other membrane separation processes, RO requires a high-pressure and high-energy input due to slow flow rates. Energy consumption accounts for around 50% of the cost of the process and consequently plays a substantial role in greenhouse gas emissions [30]. The popularity of pressure-driven membrane systems in commercial applications has been substantially limited by the energy need. Pollutant precipitation also lowers membrane durability and functionality as well as causing fouling and blockage [31]. Moreover, RO makes water more acidic because it cannot remove volatile organic compounds and chemicals from water. Membrane separation processes are also less durable and unable to self-clean, necessitating the use of different treatment methods for cleaning and recycling [32]. This has prompted scientists to come up with new membranes, such as carbon nanotubes (CNTs), for low-cost water filtration and desalination methods [33]. In addition to RO, various other water purifying processes have also been employed in the past. These techniques commonly involve ion exchange, adsorption, electrolysis, solvent extraction, precipitation, distillation, evaporation, crystallization, and ultra-, micro- and nano-filtration technologies [34]. Most of these water purification techniques consume a high amount of energy, therefore prohibiting their utility for large-scale commercial applications. With respect to this, membrane filtration has become the center of attention due to the features this technology offers [35]. The fact that there is no need for additives and thermal sources in membrane technology makes it more popular among other water purification techniques [36].

To overcome the limits of conventional membranes, innovative materials such as 2D nanosheets, carbon nanotubes and bioinspired pathways have recently attracted a lot of attention [37]. The development of remarkable transport features, such as slip flow, which might enable membranes to have ultra-high permeate flux, has stimulated similar interest in innovative materials. These materials potentially offer crucial properties for solute–solute separation, including chemical and structural consistency and easy selectivity, which are lacking in present membranes [38]. However, the addition of fabrication-induced flaws has hampered the use of these materials in membranes [39].

The nano-porous surfaces of CNTs are ideal for resisting micropollutants and ions in the liquid state. They were many times faster than previous membranes at conducting water at high rates [40]. The hydrophobic hollow shapes facilitate the smooth transportation of water without requiring any energy to drive water across the hollow tubes. The cytotoxic properties of CNT-based membranes reduce biofouling and enhance membrane lifespan by killing and eliminating microorganisms. On the other hand, pressure is required to drive water molecules through the dense porous structures of other conventional membranes such as RO. However, CNT-based membranes can replace all other conventional membranes with minimal or no energy use [41,42]. Salts are rejected and ions are retained by the nanoscale pore diameter [43].

CNTs are used for the development of water purification membranes with unique characteristics [44,45]. The permeability of water through CNTs is very high because CNTs have tube-like structures comprised of cylindrical graphene sheets [46]. The ultra-high transportation of molecules of water through these extremely narrow molecular tubes is due to the exceptionally large aspect ratio, molecularly smooth hydrophobic graphitic walls, and the nanoscale internal diameters of CNTs. Water molecules flow quicker through nanotube pores than through other pores of equivalent size. The thermodynamic and transportation parameters of limited water vary considerably from those measured in the bulk due to the smaller diameter of CNTs [42]. Previously, CNTs have been directly employed for the desalination of water [47,48,49] and used for the filtration of contaminants that slow down the desalination process [50]. CNTs require low operating power for the purification of water, which make them very attractive for water desalination. The smooth inner structure of CNTs enables them to provide an uninterrupted flow of water, blocking the contaminants [51,52].

CNT-based membranes have evolved as an appropriate water filtration technology, despite the fact that each membrane separation has its own set of advantages and disadvantages. It has shown groundbreaking results and has the ability to be commercially successful in the near future. Water permeate flux, desalination ability, solute separation, durability, antimicrobial activity, low energy consumption, cost of materials, scalability, and integration with industrial applications are all characteristics that must be met before CNT membranes become commercially available [53]. So far, microbial removal efficiencies on CNTs have outperformed any other commonly available adsorption medium [54].

Membrane technology is gaining attention because membranes have the ability to block various types of contaminants such as heavy metal ions, and organic and inorganic impurities, and act as a strong barrier in the regrowth of microorganisms. Using membrane separation enhances flow rates dramatically and almost completely reduces diffusion constraints [55]. Microbial regrowth is a major concern with water purification membranes [56,57]. Bacteria create a layer on the membrane’s surface due to their fast multiplication rate followed by further multiplication of bacterial cells. The unfavorable impacts of this phenomenon include large amounts of energy consumption during the water purification process, decreases in water flux, and the decomposition of membranes. The biodegradation of membranes is a major issue which needs serious attention. To prevent the growth of microorganisms on membrane surfaces, more efficient and robust approaches are required [58,59,60,61,62,63].

Currently, CNTs can be synthesized on a large scale by various techniques such as arc discharge processes [64], CVD [65], and photoablation/laser ablation [66]. For the development of CNT-based water purification membranes, techniques such as vertically aligned CNTs [67,68], and multi-stacked and mixed-matrix CNT membranes are commonly used. Because of their simple synthesis process, mixed-matrix CNT membranes are employed all over the world [69,70,71]. A variety of nanoparticles with wide ranging properties are available. Among these, commercially available Ag nanoparticles have received special interest because of their remarkable electrical, antibacterial and optical capabilities [72,73]. Studies have reported that silver nanoscale particles also act as a binder to bond the carbon nanotubes together [74]. Silver nanoparticles (AgNPs) have been utilized to extract lipopolysaccharides (LPS) from a water mixture, which cause inflammatory reactions, resulting in tissue injury, with up to 97 percent effectiveness [75]. AgNPs can also diminish the regrowth of microorganisms by damaging their cell membranes during the water purification process [76,77].

## 2. Materials and Methods

MWCNTs of purity > 90%, which were used in this study, were provided by Times Nano, China. The MWCNTs had an outside diameter of 10–20 nm and a length of 10–30 µm. Silver nitrate (AgNO_3_) of purity ≥ 99% was procured from Duksan Pure Chemicals, Korea, and was utilized as a salt for silver.

**Figure 1:**
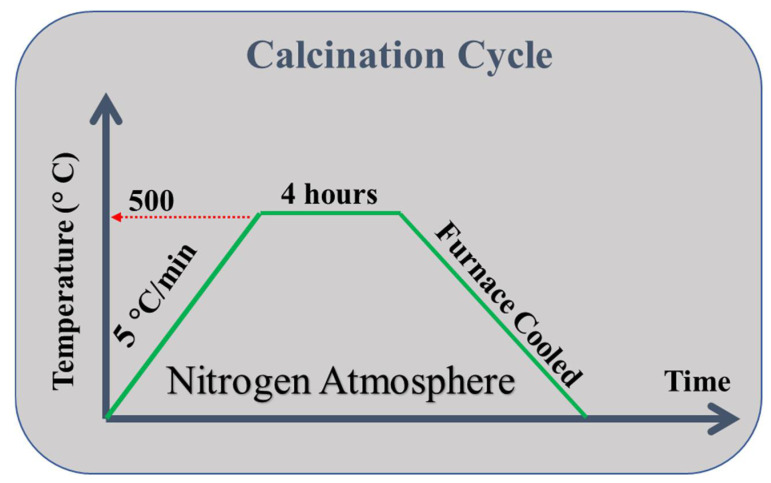
Calcination cycle explaining calcination of dried powder at 500 °C for 4 h under nitrogen atmosphere.

The surfaces of MWCNTs were impregnated with Ag nanoparticles. An amount of 0.01 g of pure AgNO3 was dispersed in 60 mL of ethanol (99.9% purity) for 1 wt% Ag loading, and 0.623 g of MWCNTs was dispersed in 300 mL of ethanol. Separately, both solutions were sonicated in an ultrasonic probe sonicator for 1 h, after which both the solutions were mixed. This was followed by post-sonication for 2 h [74]. This resulted in a homogeneous dispersion of particles, decreasing the chances of lump formation in the liquid. This mixture was then dried at 100 °C for 24 h for the evaporation of ethanol. The remaining mixture was then calcinated for 4 h at 500 °C under a nitrogen atmosphere as shown in Figure 1 to impregnate MWCNTs with Ag. This resulted in the homogeneous dispersion of Ag in the MWCNTs. This was the technique used to develop MWCNTs doped with 1 wt% Ag.

**Figure 2:**
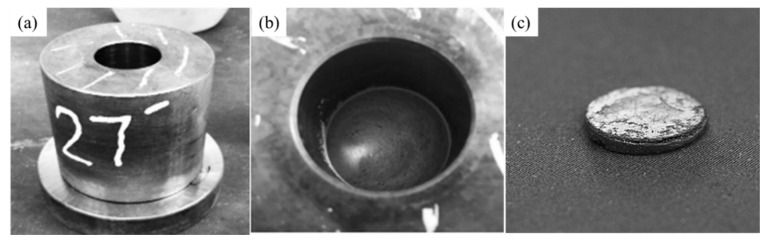
(**a**) Stainless-steel die of 27 mm diameter; (**b**) powder mixture within the die; (**c**) membrane synthesized via compaction process.

**Figure 3:**
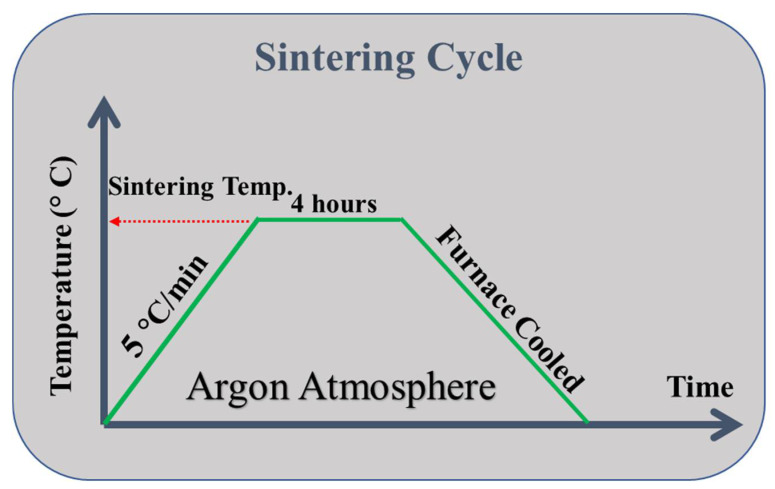
Sintering cycle describing the sintering of membranes at a rate of 5 °C/min for 4 h in an argon atmosphere at two separate temperatures of 800 °C and 900 °C.

MWCNTs impregnated with Ag nanoparticles were uniaxially compressed in a stainless-steel die with a diameter of 27 mm at two different pressures, 80 MPa and 120 MPa, as illustrated in Figure 2a,b. This resulted in membranes of approximately 27 mm diameter and 2.5–3 mm thickness, as shown in Figure 2c, having 1 wt% Ag loading. To prevent the oxidation of the MWCNTs, these membranes were sintered for 4 h in a horizontal sintering tube furnace at two different temperatures, 800 °C and 900 °C, with a heating rate of 5 °C/min in an argon atmosphere. Figure 3 depicts the sintering process.

The characteristics of doped and raw MWCNTs were investigated using a number of characterization techniques. The raw powder mixture and developed membrane samples were observed using a field emission scanning electron microscope (Lyra 3, Tescan, Brno, Czech Republic). A thermogravimetric analysis was performed with a thermogravimetric analyzer in air at a heating rate of 10 °C/min. Using an X-ray diffractometer (Rigaku MiniFlex X-ray diffractometer, Tokyo, Japan), the X-ray diffraction peaks were observed at a rate of 2°/min throughout a range of 10° to 80° (2 theta). The membranes were placed between two flat plates and subjected to a diametrical compression test on a universal testing machine. Porosity of membranes was calculated using Equation (1).

(1) $$\;Porosity=\frac{W_{wet}-W_{dry}}{\rho \times V}\times 100$$

where *W_wet_* (g) and *W_dry_* (g) are the wet and dry weights of samples, respectively, *ρ* (g/cm^3^) is the distilled water density, and *V*(cm^3^) is the sample volume. This volume is calculated using Equation (2), in which *d*(cm) is the diameter and *h*(cm) is the thickness of sample.

(2) $$V=\frac{\pi }{4}\left(d^{2}h\right)$$

**Figure 4:**
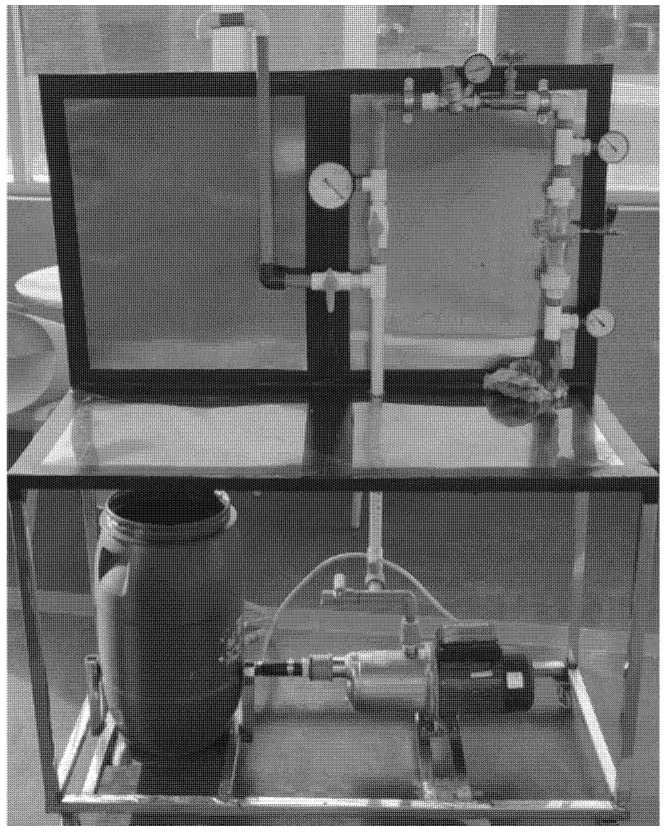
Flow loop test bench.

The dry weight of the membrane sample was determined using a density balance, and the wet weight was determined by soaking it in distilled water. The experiment was carried out five times, with the average value utilized to minimize the experimental error. The water movement across the membrane generally relies on the porosity of the membrane sample, with a higher water permeability for the membranes having higher porosity. On the flow loop test bench depicted in Figure 4, water flux measurements were taken.

**Figure 5:**
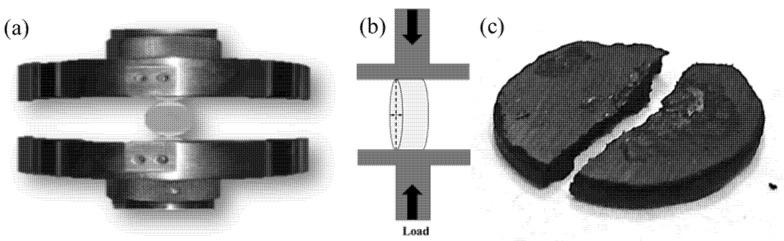
(**a**) Membrane mounted vertically between two flat plates of machine; (**b**) membrane squeezed along the diameter by applying external load; (**c**) failure of membrane across the diameter.

The pressure range across the membrane to measure the water flux was varied from 7 to 40 psi. Equation (3) was used to calculate the water flux.

(3) $$J=\frac{V}{A\times T}$$

where *J* is the pure water flux (L/(m^2^·h)), *V* denotes the volume of water that flows through the sample in liters L), *A* denotes the membrane’s cross-sectional area in meters squared (m^2^), and *T* denotes the time it takes for the water to move across the sample in hours (h) [78]. A diametrical compression test was used to determine the mechanical strength of Ag-doped MWCNT membranes. The purpose of this test was to determine the durability of porous MWCNT-Ag membranes. In a diametral compression test, the membrane was squeezed along the diameter between two flat plates of the machine as shown in Figure 5a,b, which caused tensile strains in the transverse direction. As shown in Figure 5, the membrane ruptured into two pieces as a result of tensile failure.

Equation (4) was used to calculate the diametral compressive strength in which *p* indicates the applied load and *t* indicates thickness of the membrane.

(4) σ=2P/πdt

By dropping water on the surface of MWCNTs and MWCNTs-Ag membranes, the water contact angle was determined. An arsenic removal test was carried out to assess the heavy metal removal efficiency of synthesized membranes. The adsorption capacity of the selected CNT-1 wt% Ag (800 °C/120 MPa) membrane was evaluated using the flow loop system shown in Figure 4. Three membranes each having a 27 mm diameter and a 3 mm thickness were subjected to the test at three different cross-membrane pressures of 10, 15 and 20 psi, respectively. Using an arsenic standard solution (1000 ppm), a solution with an initial arsenic concentration of 2 ppm was prepared. A 1M KOH solution was used to maintain the pH of solution at 5.8. The filtrate (10 mL) was collected after a single run through the membrane and analyzed using an Optical Emission Spectrometer (OES). The OES testing facility of Pakistan Council of Research in Water Resources (PCRWR) was outsourced to determine the arsenic concentration in the pre- and post-purification conditions.

## 3. Results and Discussion

### 3.1. Characterization of Raw and Impregnated MWCNT Powder

#### 3.1.1. FESEM and EDX Analysis

**Figure 6:**
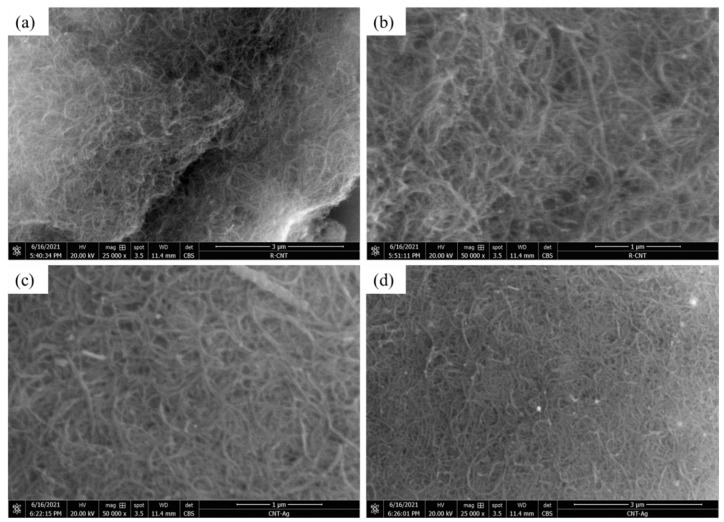
FESEM images of (**a**) raw MWCNTs at low magnification exhibiting a clot-like appearance; (**b**) high-magnification image of raw MWCNTs; (**c**) high-magnification image of MWCNTs-Ag showing effective sonication; (**d**) low-magnification image of MWCNTs-Ag.

**Figure 7:**
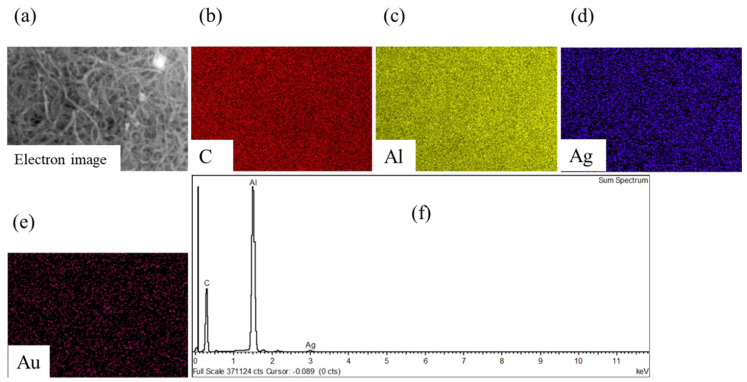
(**a**) Electron beam microscopy image of MWCNT-Ag powder; (**b**) signal of carbon from MWCNTs; (**c**) signal of aluminum from sample holder; (**d**) uniform dispersion of Ag particles within the MWCNT network; (**e**) signal of gold from conductive coating; (**f**) EDX spectrum showing dispersion of Ag particles with MWCNT powder.

Figure 6 depicts the FESEM micrographs of raw and Ag-impregnated MWCNTs. At low magnification, the MWCNTs were more cluttered, exhibiting a clot-like appearance (Figure 6a), while a relatively rough appearance was observed at high magnification (Figure 6b). A smoother and less entangled appearance of the MWCNTs-Ag at high magnification indicated that sonication was effective (Figure 6c), while at low magnification it was difficult to assess the distribution of Ag particles within the MWCNT network (Figure 6d). The EDX map acquired from MWCNT-Ag powder, shown in Figure 7a, indicates a uniform dispersion of Ag particles (Figure 7d) within the MWCNT network (Figure 7b). The signal of aluminum metal (Figure 7c) is captured from the sample holder, while the Au signal (Figure 7e) is attributed to the conductive coating. The EDX spectrum is shown in Figure 7f.

#### 3.1.2. Thermal Degradation Analysis

**Figure 8:**
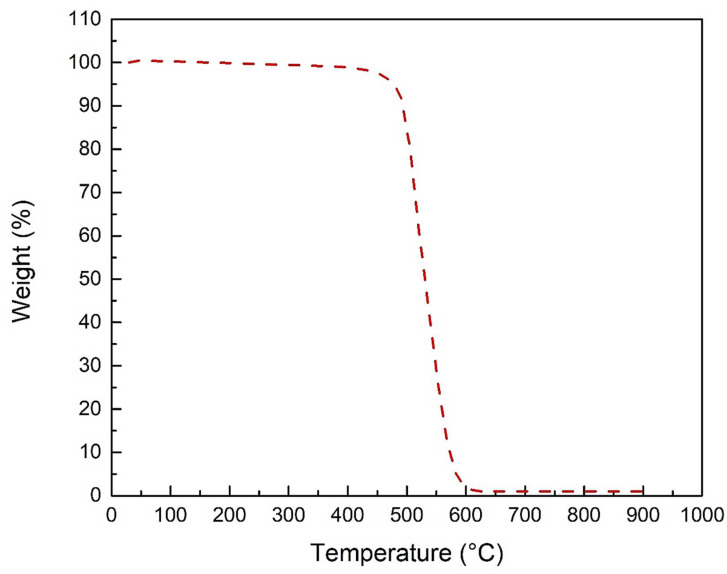
Thermogravimetric analysis of MWCNTs-Ag.

The thermo gravimetric analysis curve for MWCNTs impregnated with 1%Ag loading is shown in Figure 8. For MWCNTs-1% Ag, the initial degradation temperature was 458 °C and, at around 600 °C, total degradation was noticed. The weight percent of the sample was decreased to approximately the percentage of Ag loading at 900 °C, which was confirmed.

#### 3.1.3. X-Ray Diffraction (XRD)

**Figure 9:**
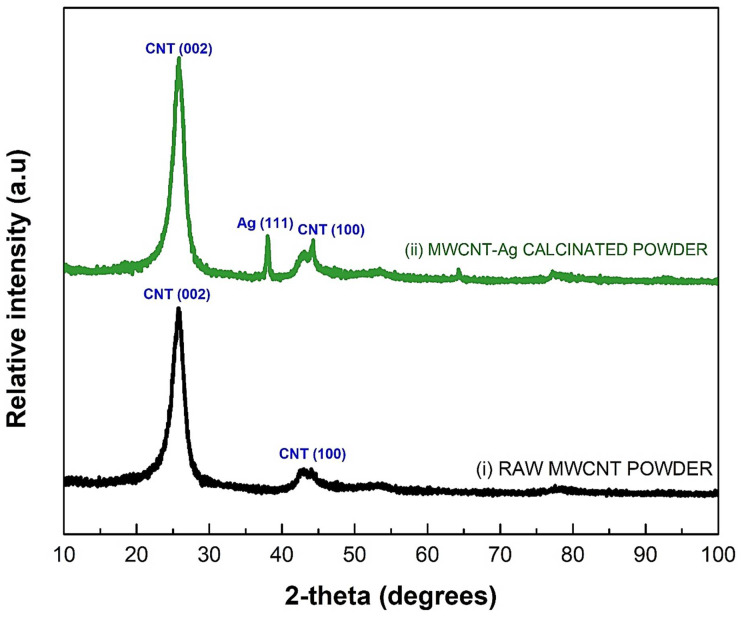
X-ray diffraction pattern for (**i**) raw MWCNT powder; (**ii**) MWCNT-Ag powder.

Figure 9i shows the XRD pattern of raw MWCNTs, which shows two prominent distinctive peaks at 2θ∼25 and 44 degrees, which correlate to the hexagonal graphite lattice of MWCNTs. For MWCNTs-Ag, peaks of Ag were detected at 2θ∼38°, as shown in Figure 9ii. After the calcination of MWCNT-Ag powder, crystals of Ag were formed, which were detected in XRD peaks.

### 3.2. Membrane Characterization

#### 3.2.1. Field Emission Scanning Electron Microscopy (FESEM)

**Figure 10:**
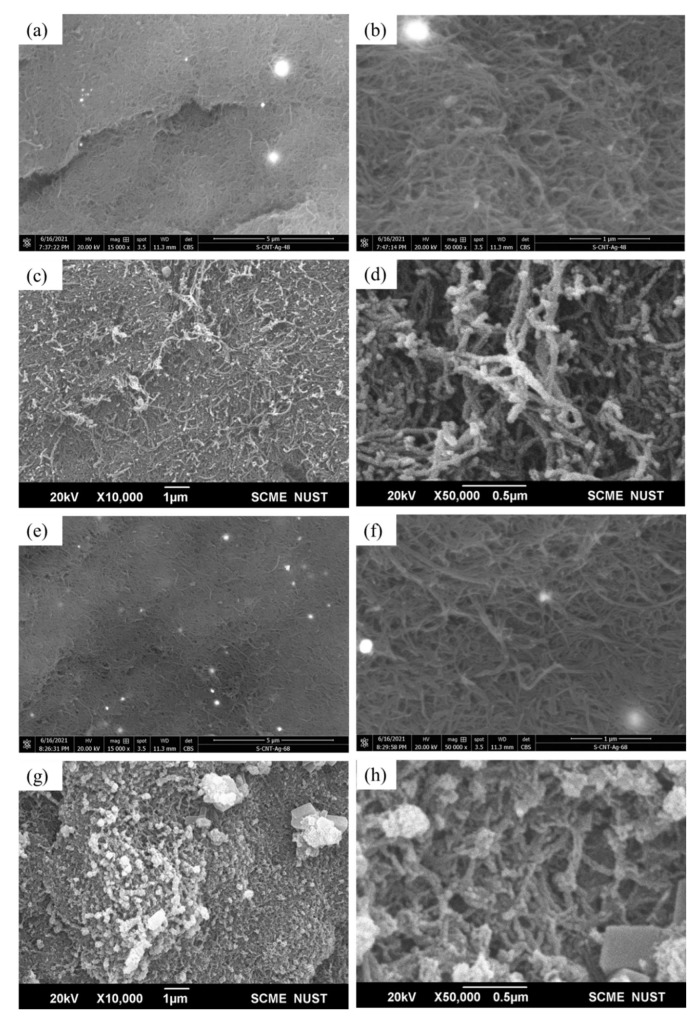
FESEM images of sintered membranes. (**a**) Low-magnification image of MWCNT-Ag membrane synthesized at 800 °C and 80 MPa; (**b**) high-magnification image of 800 °C and 80 MPa membrane; (**c**) image of membrane (900 °C and 80 MPa) at low magnification; (**d**) high-magnification image of 900 °C and 80 MPa membrane; (**e**) low-magnification image of membrane produced at 800 °C and 120 MPa; (**f**) high-magnification image of 800 °C and 120 MPa membrane; (**g**) Image of 900 °C and 120 MPa membrane at low magnification; (**h**) high-magnification image of membrane synthesized at 900 °C and 120 MPa.

Figure 10 illustrates FESEM images of sintered membranes of MWCNTs doped with Ag nanoparticles. It shows that Ag nanoparticles are evenly dispersed on the surface of membranes. Pores in the membrane are clearly visible in samples sintered at 800 °C (Figure 10b). FESEM images of MWCNT-Ag membranes (800 °C; 80 MPa) show visible cracks at low magnification with bright spots of Ag particles (Figure 10a), while there was no agglomeration of particles at high magnification, which highlights the effective dispersion of Ag within the MWCNTs (Figure 10b). A low-magnification image (Figure 10c) of (900 °C; 80 MPa) sample shows the homogeneous dispersion of Ag particles on the surface of membranes, while at high magnification, bright knots of Ag particles are clearly visible within the MWCNT network (Figure 10d). On the other hand, the MWCNT-Ag membrane synthesized at 800 °C and 120 MPa illustrates a smooth crack-free surface at low magnification (Figure 10e) with bright spots of Ag particles at high magnification (Figure 10f). Figure 10g shows a visible agglomeration of Ag particles for samples synthesized at 900 °C and 120 MPa, indicating an inhomogeneous sonication of the powder (Figure 10h). However, a crack-free surface without visible porosity is obvious. It is pertinent to point out that Ag has a significant effect on the attributes of membranes and binding together of the MWCNTs.

#### 3.2.2. Porosity

**Figure 11:**
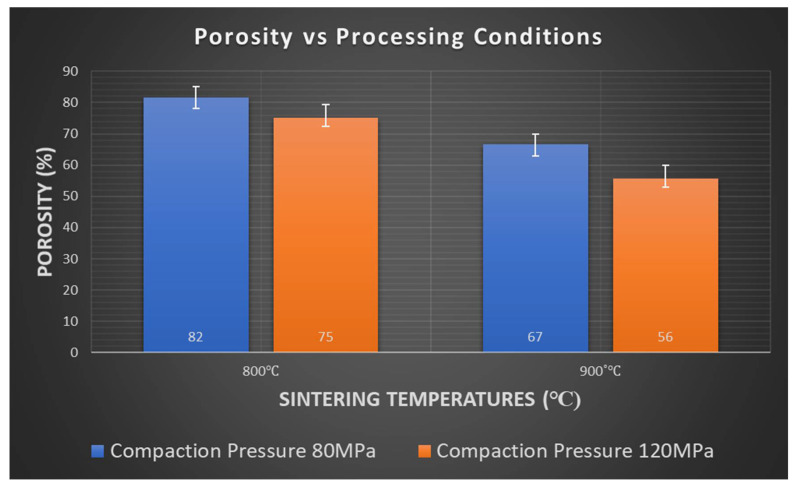
Variation in porosity with respect to initial compaction load and sintering temperatures.

Porosity was found to decrease as the initial compaction load and sintering temperature increased. A maximum porosity of 82% was achieved in the sample prepared at 800 °C with a compaction load of 80 MPa. A minimum porosity of 56% was achieved in the sample prepared at 900 °C with a compaction load of 120 MPa (Figure 11). The effect of temperature was significant towards a better densification, primarily due to higher diffusion of Ag within the MWCNT network and wider coverage of voids present between the MWCNT network by Ag particles. Additionally, better adhesion of MWCNT-Ag-MWCNT was realized because of the higher activation energy available for bonding.

#### 3.2.3. Water Flux Measurement

**Figure 12:**
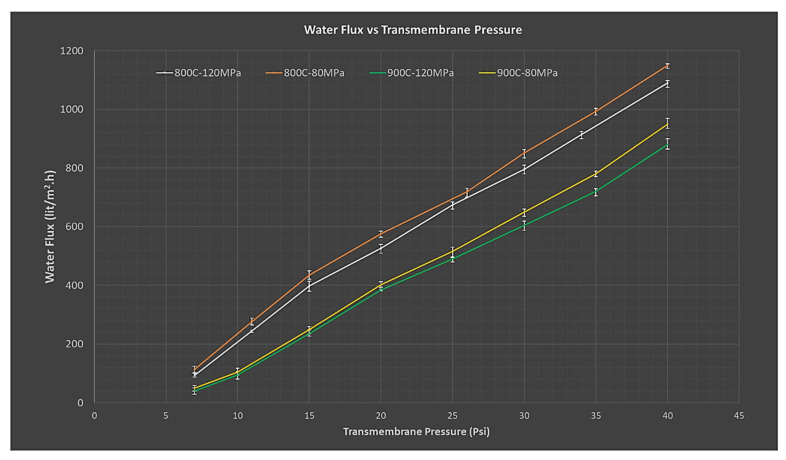
Water permeate flux as a function of initial compaction load and sintering temperatures.

Water flux values were also influenced by the initial compaction loads and sintering temperatures. Generally, higher flux was measured in more porous membranes which were compacted with low compaction loads and sintering temperatures. A linearly increasing trend in water flux was observed in all samples with an increase in pressure across membranes (7–40 Psi), as illustrated in Figure 12. All the membranes developed through a powder metallurgical route showed overall higher flux values than the ones prepared via chemical vapor deposition, a technique described in the literature [79].

#### 3.2.4. Diametrical Compression Test

**Figure 13:**
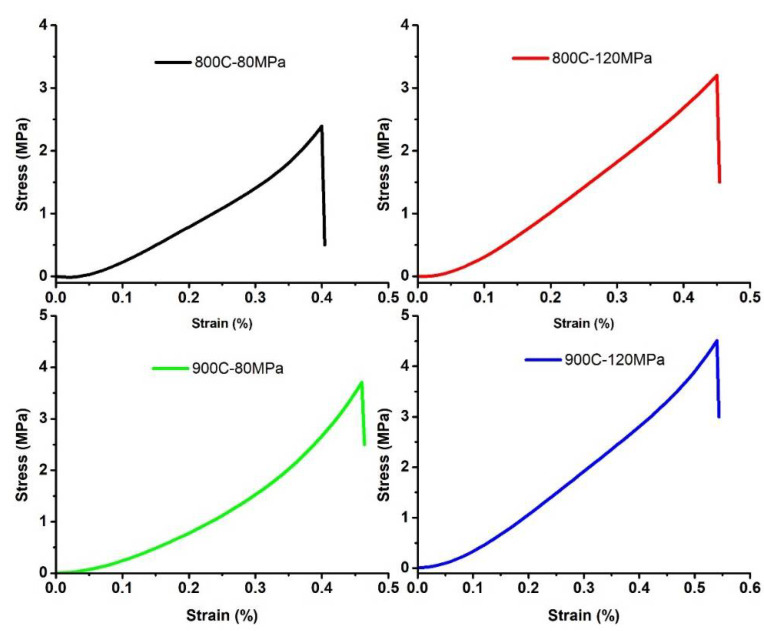
Curves of diametrical compression test.

Diametrical strength was measured to have increased with the increase in initial compaction pressure and sintering temperature. The sample synthesized at 900 °C and compacted at 120 MPa achieved the highest strength of 4.5 MPa, as shown in Figure 13. The strengths observed for the current membranes were consistent with the existing literature on diametrical compression strength for Alumina/CNT-based membranes [80].

#### 3.2.5. Hydrophilic Behavior of Raw and Ag-Doped MWCNT Membranes

**Figure 14:**
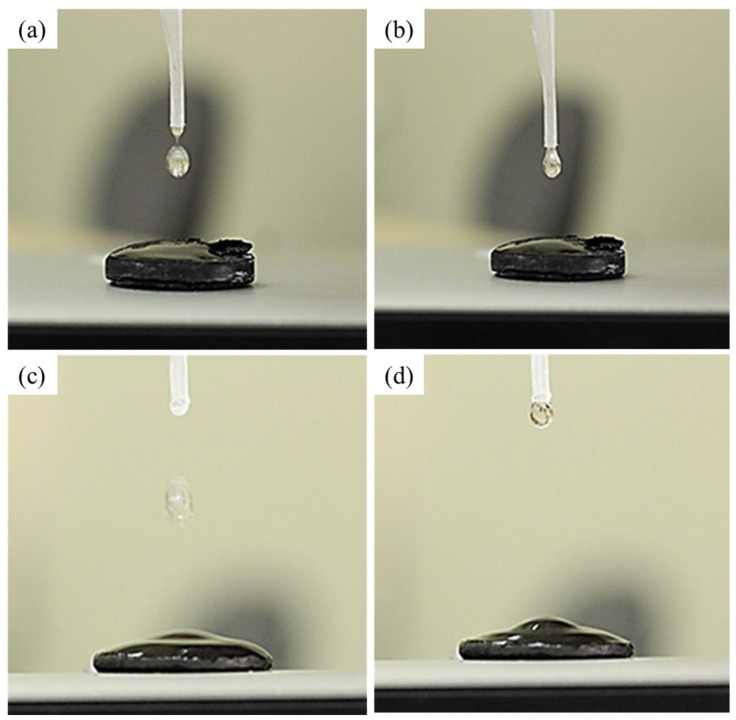
Contact angle measurement (**a**,**b**) images of raw MWCNT membranes; (**c**,**d**) images of MWCNT-Ag membranes.

The hydrophilic nature of the raw and Ag-doped MWCNTs was assessed qualitatively by dropping deionized water onto the surface of MWCNTs and MWCNT-Ag membranes and capturing the image using a digital single-lens reflex (DSLR) camera, as shown in Figure 14. To reduce the experimental error, the experiments were carried out multiple times with various membranes. The water completely spread on the surface of membranes, and it can therefore be concluded that the inclusion of Ag particles had no effect on the membrane’s hydrophilicity.

#### 3.2.6. Water Purification Capability

Using an arsenic standard solution (1000 ppm), a mixture with an initial arsenic concentration of 2 ppm was prepared. A 1M KOH solution was used to keep the pH of the solution at 5.8. An arsenic removal test was then performed to determine the MWCNT-Ag membrane’s ability to remove arsenic. The ultimate concentration of arsenic in the filtrate was equivalent to 505 ppb (average value of three membranes), indicating that the experiment was successful in removing 75% of the arsenic. The experiment was repeated three times to reduce experimental error, with the average value being reported. However, the membranes developed in the current study can be further utilized for rigorous water purification experiments to investigate the selectivity of heavy metals via batch adsorption analysis.

## 4. Conclusions

Ag-doped MWCNT membranes were developed successfully. The membranes showed unique characteristics and were notably influenced by sintering temperature and compaction load. Silver particles also acted as a binding agent for MWCNTs. As verified by the contact angle measurement, there was no change in the hydrophilic nature of membranes. The porosity of the membranes was noticed to decrease as the compaction load and sintering temperature increased due to a higher diffusion of Ag within MWCNTs and extensive coverage of voids. The mechanical strength of the membranes was measured to rise as the sintering temperature and compaction load increased, as confirmed by the diametrical compression test. Higher values of water permeate flux were measured in membranes compacted at low compaction pressures and sintering temperatures. The membranes were found to have a profound influence on the elimination of arsenic from the standard arsenic solution.

## Data Availability

Not applicable.
